# Will HIV Vaccination Reshape HIV Risk Behavior Networks? A Social Network Analysis of Drug Users' Anticipated Risk Compensation

**DOI:** 10.1371/journal.pone.0101047

**Published:** 2014-07-03

**Authors:** April M. Young, Daniel S. Halgin, Ralph J. DiClemente, Claire E. Sterk, Jennifer R. Havens

**Affiliations:** 1 Department of Epidemiology, University of Kentucky, Lexington, Kentucky, United States of America; 2 Center on Drug and Alcohol Research, University of Kentucky, Lexington, Kentucky, United States of America; 3 LINKS Center for Social Network Analysis, Gatton College of Business and Economics, University of Kentucky, Lexington, Kentucky, United States of America; 4 Department of Behavioral Sciences and Health Education, Emory University, Atlanta, Georgia, United States of America; Columbia University, United States of America

## Abstract

**Background:**

An HIV vaccine could substantially impact the epidemic. However, risk compensation (RC), or post-vaccination increase in risk behavior, could present a major challenge. The methodology used in previous studies of risk compensation has been almost exclusively individual-level in focus, and has not explored how increased risk behavior could affect the connectivity of risk networks. This study examined the impact of anticipated HIV vaccine-related RC on the structure of high-risk drug users' sexual and injection risk network.

**Methods:**

A sample of 433 rural drug users in the US provided data on their risk relationships (i.e., those involving recent unprotected sex and/or injection equipment sharing). Dyad-specific data were collected on likelihood of increasing/initiating risk behavior if they, their partner, or they *and* their partner received an HIV vaccine. Using these data and social network analysis, a "post-vaccination network" was constructed and compared to the current network on measures relevant to HIV transmission, including network size, cohesiveness (e.g., diameter, component structure, density), and centrality.

**Results:**

Participants reported 488 risk relationships. Few reported an intention to decrease condom use or increase equipment sharing (4% and 1%, respectively). RC intent was reported in 30 existing risk relationships and vaccination was anticipated to elicit the formation of five new relationships. RC resulted in a 5% increase in risk network size (n = 142 to n = 149) and a significant increase in network density. The initiation of risk relationships resulted in the connection of otherwise disconnected network components, with the largest doubling in size from five to ten.

**Conclusions:**

This study demonstrates a new methodological approach to studying RC and reveals that behavior change following HIV vaccination could potentially impact risk network connectivity. These data will be valuable in parameterizing future network models that can determine if network-level change precipitated by RC would appreciably impact the vaccine's population-level effectiveness.

## Introduction

Preventive HIV vaccines have the potential to curtail the HIV epidemic. However, many have voiced concerns that HIV vaccination could elicit increased HIV risk behavior among vaccine recipients. This phenomenon, or ‘risk compensation’, occurs when diminished perceived susceptibility resulting from participation in some preventive intervention causes a subsequent increase in risk behavior [1]. Given that the first HIV vaccines on the market are likely to be only partially effective, risk compensation could substantially dampen and, in some circumstances, offset the vaccine's public health benefit [2]–[5]. In fact, some models have predicted that with a combination of frequent risk compensation and low vaccine efficacy, an HIV vaccine campaign could actually *increase* HIV incidence [2], [3].

Findings on the hypothetical likelihood that HIV vaccinated individuals will engage in risk compensation have been mixed. In HIV vaccine acceptability studies implemented in diverse settings, participants have expressed concern that others would increase their sexual risk behavior if vaccinated [6]–[10]. However, in studies asking participants about their personal likelihood of risk compensation, fewer anticipate behavioral changes [11]–[13]. Findings from research embedded within HIV vaccine trials have generally identified no substantial increase in risk behavior during trial participation [14]–[21], though there is some evidence to the contrary [22].

The methodology employed in most HIV vaccine risk compensation studies to date has focused exclusively on individuals. Despite an abundance of evidence suggesting that social networks can play an important role in HIV and sexually transmitted infection (STI) transmission [23]–[31], HIV risk behavior [25], [32], [33], and involvement in preventive interventions [34]–[36], the HIV vaccine acceptability literature is devoid of insights into the network-level dynamics of risk compensation.

Previous individual-level studies have captured *if* and *to what degree* individuals will engage in risk compensation [e.g., [6], [37]–[39], but they have not captured *with whom*. Consequently, there is currently a gap in understanding about the ability of HIV vaccination to alter the dynamics and structure of HIV risk networks. Individual-level measures have been used to inform risk compensation parameters in mathematical models aimed at determining the efficacy required for an HIV vaccine to achieve impact on population-wide HIV incidence (e.g., male-initiated condom use [3], [40], condom use [2], [41], number of partners [5], [41], number of injections and needle sharing [42]). However, if risk compensation increases the connectivity of risk networks, the impact of risk compensation on HIV incidence may be underestimated. HIV vaccination inherently will disrupt the transmission of HIV through risk networks, but the degree of disruption will depend on behavioral changes and the network position of those who engage in risk compensation. Even minor changes in network configuration may affect epidemic potential, but improved understanding of anticipated network-level changes is needed to inform network models that can estimate the effectiveness of community HIV vaccine initiatives.

The current study used network analysis to examine drug users' risk relationships and anticipated risk compensation. The current risk network of participants was compared to a simulated "post-vaccination" risk network, constructed according to participants' anticipated behavior change (under variable hypothetical vaccination scenarios) with each of their current partners and new partners. The overarching aim of the study was to introduce a new methodological and conceptual approach for examining risk compensation in the context of HIV vaccination.

## Methods

### Sample

This study was implemented in the context of the ongoing longitudinal Social Networks among Appalachian People (SNAP) study, the methods of which have been described in detail elsewhere [43], [44]. The purpose of SNAP is to examine the epidemiology of HIV, hepatitis C, and herpes-simplex 2 among illicit drug users in a rural Appalachia in the United States. Eligibility criteria for the study included being at least 18 years of age, residing in an Appalachian county in Kentucky, and non-medical use of prescription opioids, heroin, crack/cocaine or methamphetamine to get high in the prior 30 day period. Participants (n = 503) were recruited from November 2008 to August 2010, using respondent driven sampling. Participants completed interviewer-administered questionnaires and HIV testing at baseline and every six months afterward. From March 2012 to May 2013, 435 participants completed their 24-month follow-up assessment. All participants tested HIV negative using the OraQuick *ADVANCE* Rapid HIV-1/2 Antibody Test (OraSure, Bethlehem, PA).

Following their 24-month interview, 433 participants were invited to complete an interviewer-administered questionnaire on their attitudes toward HIV vaccination and intent to change behavior if vaccinated against HIV. Two 24-month SNAP participants were not invited, as they were interviewed in jail and time-constraints prohibited the interviewers' ability to administer the questionnaire. All invited participants provided written informed consent to participate and were compensated $35 for their time. The protocol was approved by the University of Kentucky's Institutional Review Board and a Certificate of Confidentiality was obtained.

### Network data collection

The SNAP interview included a name-generator questionnaire that was used to establish drug, sex, and social support networks. The focus of the present study is the 'risk network', which consists of sexual relationships and/or relationships in which partners engaged in injection drug use together in the past 6 months. Participants gave the first name and last initial, age, and gender of each of their risk partners (a maximum of twenty-four partners could be named). The reported names and demographic information were then cross-referenced against those of others enrolled in the study to construct the network of relationships among participants (i.e. the 'sociometric network'). If the relationship could not be confirmed through the cross-referencing procedure, the community-based interviewers were consulted for their knowledge of reported relationships. If cross-referencing nor consultation of interviewers revealed a confirmed linkage, the named network member was determined to not be enrolled in the study (i.e. outside of the sociometric network).

**Figure 1:**
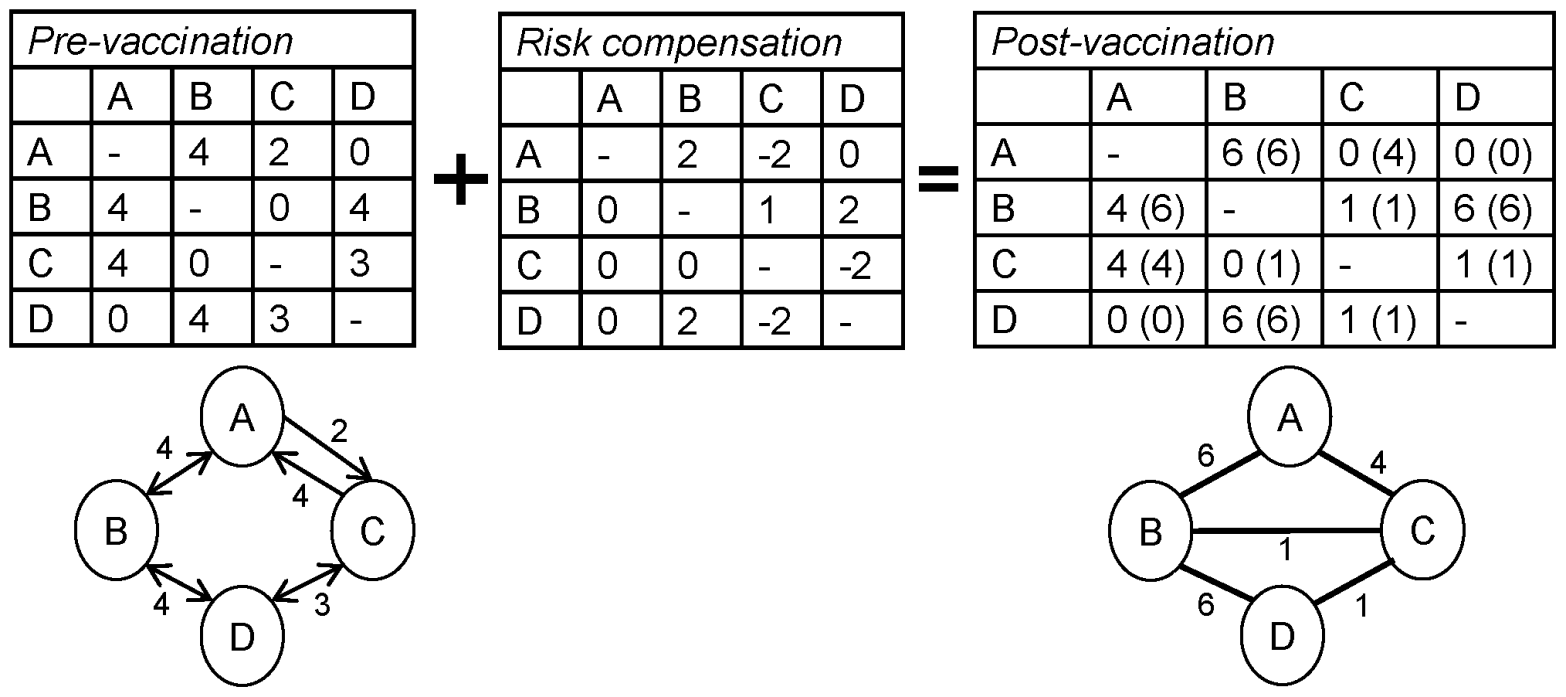
Illustration of procedure for constructing pre- and post-vaccination risk networks for comparison. Figure 1 displays a network of risk relationships among participants A, B, C and D. The corresponding adjacency matrixes are also presented. The values of the pre- and post-vaccination network ties represent frequency of HIV risk behavior, or the sum of three Likert scales on which participants rated the frequency of unprotected sex and frequency of needle and cooker sharing with the alter. Values in the risk compensation matrix represent the degree of behavior change anticipated to occur after HIV vaccination, with negative numbers representing a decrease in risk behavior, zeros representing no change, and positive numbers representing risk compensation. To construct the post-vaccination matrix, the risk compensation matrix was added to the pre-vaccination matrix. Also, Participant D reported that they would initiate a risk relationship with Participant B, so a tie was added and a one was entered in the corresponding cell of the post-vaccination matrix. Numbers in parentheses represent the symmetrized version of the network; the symmetrized version was used for analysis.

Two versions of the risk network were constructed: an *Expansive Network*, which included all named alters (study participants and non-participants), and a *Sociometric Network*, which only included relationships between SNAP participants. For analysis, each network was represented in the form of an actor-by-actor adjacency matrix, ***A***_ij_ (example shown in Figure 1). Network analysis and visualization were conducted using UCINET (version 6) [45] and NetDraw (version 2) [46].

#### Risk Behavior

For the present analyses, four behavioral networks were constructed. One network contained valued data representing the current frequency of HIV risk behavior; the values represented the sum of three Likert scales on which participants rated the frequency of any unprotected sex (0 = always use condoms, 1 = use condoms half the time, 2 = use condoms less than half the time, 3 = never use condoms) and frequency of needle and cooker sharing (each measured on 4-point scales: 0 = none, 1 = less than once per month, 2 = monthly, 3 = weekly, 4 = daily) with the alter in the past 6 months. Thus, the value for the summed risk behavior scale could range from 0 to 11. This network was considered to be the "**pre-vaccination network**", as it represented risk behavior in the absence of an HIV vaccine. Of note, the pre-vaccination network was ‘symmetrized’ for analysis. Symmetrized networks do not take into account *who* reported the information; for example, if one person reported sharing works with an alter, the relationship was presumed to be reciprocal. All data (binary, ordinal and continuous) were symmetrized by taking the maximum value reported for each relationship.

#### Risk Compensation

For each sex partner and/or partner with whom drug injection equipment was shared, respondents were asked about their likelihood of increasing risk behavior if they, their partner, or both they and their partner received an HIV vaccine. Specifically, respondents were asked three items to assess sex-related risk compensation, "If [you/alter/you and alter] got an HIV vaccine that was 90% effective, would you use a condom with them… [‘Much less often' (+2), ‘Less often' (+1), ‘More often' (−1), ‘Much more often' (−2), ‘We wouldn't change how often we used a condom' (0)]". Respondents were also asked three injection-related items, "If [you/alter/you and alter] got an HIV vaccine that was 90% effective, would you share injection equipment…" [‘Much less often' (−2), ‘Less often' (−1), ‘More often' (+1), ‘Much more often' (+2), ‘We wouldn't change how often we shared equipment' (0)]. Participants were also asked about alters' HIV status.

Respondents were also given the option to name new individuals with whom they would initiate risk behavior if they received the HIV vaccine. Specifically, respondents were asked, “Imagine that you got an HIV vaccine that was 90% effective. Is there anyone else you can think of who you may start [having sex/sharing works] with? For example, [list of all social support, drug, and sex network members named in the SNAP interview].” Respondents then gave the name (first and last initial), age, and gender of each individual. These data were cross-referenced using the same procedures described above to determine if the new relationship was with someone participating in the SNAP study. Each new relationship was conservatively assigned a value of "1" in the post-vaccination network (described below).

The symmetrized **post-vaccination network** was constructed using the risk compensation data. The maximum values from the three sexual and injection-related risk compensation questions were added to the Likert scale ratings given for the dyad's current unprotected sex and equipment sharing behavior, respectively. The resulting condom use and equipment sharing ratings were then summed to produce a valued "post-vaccination network" representing each dyad's frequency of risk behavior in the presence of HIV vaccination. An example of this process is shown in Figure 1. Note that the post vaccination matrix was symmetrized by taking the maximum value reported for each relationship, as demonstrated by the numbers in parentheses in Figure 1.

### Statistical Analyses

To examine changes that may occur to the overall risk network structure in the presence of HIV vaccination, symmetrized versions of the pre-vaccination and post-vaccination risk networks were compared. For each network, structural measures of network size, cohesiveness (diameter, component structure, density, and k-cores) and centrality were computed. Each of these measures were chosen a priori based on evidence that they can play a role in network-level HIV and STI transmission and related behaviors in risk networks [23], [24], [26], [27], [32], [47]–[50]. Network size, or *diameter*, is the length of the longest path in the network [51]. *Components* are network structures within which all individuals are connected directly or indirectly through at least one path [52]. *Isolates* are participants who are disconnected from everyone in the network. *Density*, for binary matrices, is the number of connections in the network reported as a fraction of the total connections possible. For valued data, density represents the average value of relationships within the network [52]. The density of the two networks was compared by using a bootstrap paired sample t-test conducted in UCINET. The paired sample t-test of density on the valued networks determined if there was a difference in the mean overall tie strengths of the pre- and post-vaccination networks [52].

*Network Centralization* [53], based on computation of degree centrality [53], represents the degree to which the networks are centralized around one or a few actors [54]. The centralization value, which ranges from 0 to 1, reflects the extent to which all network members are connected through one central actor (i.e. visualized in the shape of a star) [51], [53]. Higher values of centralization are indicative of more hierarchy [54]. Finally, *k-cores* capture information on participants' location within cohesive risk network subgroups. A *k*-core is a maximal subgroup of individuals within a network that are all connected to at least *k* other members in the group. For example, a 2-core refers to a group of two or more people who are connected to at least two other members of the group [49]. Two-cores are hypothesized to be conducive to HIV and STI transmission [26], [49]; thus, for the present analysis, networks were compared in terms of the number of 2-cores present in the network.

Of note, most indices required dichotomization of valued data; however, degree centrality, centralization, and density could also be computed on valued data. For these three indices, the valued and binary comparisons are presented.

## Results

Participants were predominantly White (94%), 45% were female, and only 25% were married. The median age of participants was 34 years (range: 21–68). Slightly over half (58%) had graduated from high school, 39% were unemployed, and the median monthly income (from all sources) was $698. Most (82%) reported at least one sexual partner in the past 6 months, 24% reported having multiple partners, 71% reported unprotected sex with at least one partner in the past 6 months, and 20% reported unprotected sex with a person who injects drugs in the past 6 months.

**Figure 2:**
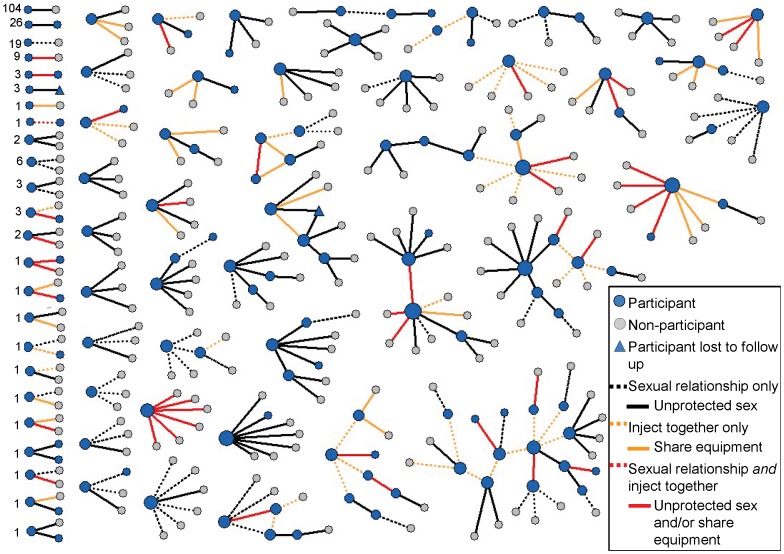
Sexual and injection-related risk networks of respondents and named alters. Nodes are sized by degree centrality (i.e. number of partners).

**Figure 3:**
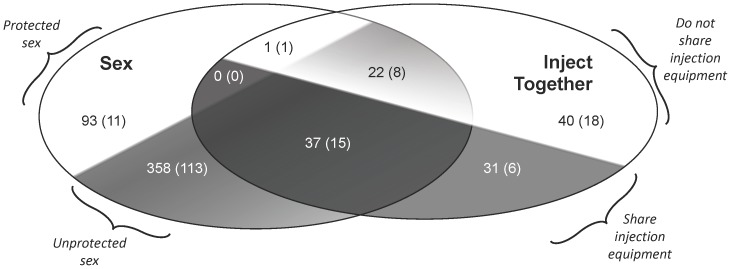
Risk relationships in the expansive and sociometric networks. Number of sexual and injection relationships in the expansive network, with numbers in parentheses indicating the subset of relationships present in the sociometric network (i.e. confirmed ties). Relationships indicated in the shaded portions of the figure (e.g., unprotected sex and equipment sharing) comprise the expansive and sociometric pre-vaccination networks.

The risk network is shown in Figure 2. Of the 433 participants, 353 reported at least one sexual relationship and 45 reported a relationship that involved sharing drug injection equipment. All alters were reported to be HIV negative. Overall, the symmetrized network contained 458 sexual relationships (two involving sex between two men), 368 of which involved unprotected sex. The network included 65 relationships that involved equipment sharing, including 34 that involved equipment sharing *and* unprotected sex. Figure 3 displays the overlap between the injection and sexual relationships and displays the number of sociometric ties (in parentheses) relative to the number of expansive network ties.

### Risk compensation in current relationships

**Figure 4:**
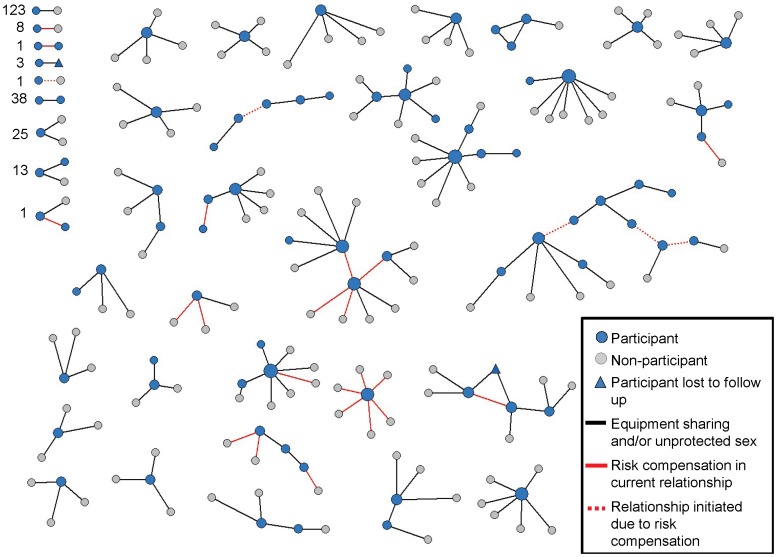
Risk compensation within a risk network of rural drug users. Nodes are sized by degree centrality (i.e. number of partners). The figure does not include the 95 participants who did not someone with whom they shared equipment or had unprotected sex.

Figure 4 shows relationships involving intended risk compensation (shown as red lines). There were 30 relationships in which the respondent reported a likelihood of risk compensation, including three that would involve increased equipment sharing and 27 that would involve increased unprotected sex (there were no relationships involving intent to increase equipment sharing *and* unprotected sex). There were some individuals who would increase their sexual risk behavior with many partners, including one person that reported risk compensation for six sexual relationships and another who reported it for four relationships. Overall, sexual risk compensation resulted in the addition of fourteen relationships to the risk network (i.e. individuals who previously always used condoms would begin having unprotected sex); the other sixteen relationships involving intended risk compensation occurred within relationships already involving either unprotected sex or equipment sharing. Of note, reported intention to *increase* condom use after HIV vaccination resulted in the removal of four relationships in the risk network.

As shown in Table 1, which describes responses to the risk compensation questions, the likelihood of risk compensation did not vary substantially by vaccination scenario (i.e. vaccination of self, partner, or of self and partner). Sexual risk compensation was intended in only 5.3% of sexual relationships in the network and risk compensation related to equipment sharing was only intended in 4.4% of equipment sharing relationships. Interestingly, condom use was intended to *increase* after HIV vaccination in 4.7% of sexual relationships. Overall, the vast majority of participants reported they would not change their sexual or injection-related risk behavior under any vaccination scenario (91.2% and 93.3%, respectively).

**Table 1:** Intent to engage in sexual and injection-related risk compensation in three HIV vaccination scenarios.

	Vaccination of self		Vaccination of partner		Vaccination of self and partner		Total	
	Egos n(%)^a^	Dyads n(%)^b^	Egos n(%)^a^	Dyads n(%)^b^	Egos n(%)^a^	Dyads n(%)^b^	Egos n(%)^a^	Dyads n(%)^b^
*Change in condom use*								
**Much less often**	6 (1.7)	8 (1.6)	6 (1.7)	8 (1.6)	6 (1.7)	8 (1.6)	7 (2.0)	9 (1.8)
**Less often**	10 (2.8)	17 (3.3)	10 (2.8)	17 (3.3)	10 (2.8)	18 (3.5)	10 (2.8)	18 (3.5)
More often	13 (3.7)	18 (3.5)	11 (3.1)	16 (3.1)	12 (3.4)	17 (3.3)	13 (3.7)	18 (3.5)
Much more often	6 (1.7)	6 (1.2)	6 (1.7)	6 (1.2)	6 (1.7)	6 (1.2)	6 (1.7)	6 (1.2)
Would not change	324 (91.8)	460 (90.2)	326 (92.4)	462 (90.6)	324 (91.8)	460 (90.2)	322 (91.2)^c^	458 (89.8)^c^
*Change in equipment sharing*								
Much less often	0 (0.0)	0 (0.0)	0 (0.0)	0 (0.0)	0 (0.0)	0 (0.0)	0 (0.0)	0 (0.0)
Less often	0 (0.0)	0 (0.0)	0 (0.0)	0 (0.0)	0 (0.0)	0 (0.0)	0 (0.0)	0 (0.0)
**More often**	2 (4.4)	2 (2.9)	2 (4.4)	2 (2.9)	2 (4.4)	2 (2.9)	2 (4.4)	2 (2.9)
**Much more often**	1 (2.2)	1 (1.5)	1 (2.2)	1 (1.5)	1 (2.2)	1 (1.5)	1 (2.2)	1 (1.5)
Would not change	42 (93.3)	65 (95.6)	42 (93.3)	65 (95.6)	42 (93.3)	65 (95.6)	42 (93.3)^c^	65 (95.6)^c^

**Bold** indicates responses consistent with intent to engage in risk compensation.

a Percentages are based on the total number of participants reporting at least one sexual (n = 353) or equipment-sharing relationship (n = 45). Of note, 31 people reported a relationship with someone with whom they had sex *and* shared injection equipment. The total number of respondents is greater than 367 because each respondent could give a different response for each named alter.

b Percentages for rows corresponding to changes in condom use and equipment sharing are based on the total number of sexual (n = 511) and equipment-sharing relationships (n = 68), respectively.

c Number of respondents and dyads in which no change was reported under *all* three vaccination scenarios.

### Risk compensation involving initiation of new risk relationships

On the open-ended questions, four respondents listed specific people with whom they would begin having unprotected sex (n = 3) and/or sharing equipment (n = 1). Three respondents gave first names and last initials of a total of four individuals who were confirmed to be in the study, and one person named someone not in the study.

### Structural changes to the risk network due to risk compensation

Descriptive comparisons of the pre-vaccination and post-vaccination networks are shown in Table 2. The expansive post-vaccination network contained fifteen more relationships and fifteen fewer isolates than the pre-vaccination network. In both the expansive and sociometric networks, diameter of the post-vaccination network was twice that of the pre-vaccination network. The size of the main component increased from 14 to 16 due to risk compensation; however, the overall average component size remained similar (2.63 and 2.70, respectively). The average degree centrality and the centralization of the post- and pre-vaccination network were also similar.

**Table 2:** Comparison of pre- and post-vaccination risk networks.

		Expansive Network		Sociometric Network	
Characteristic		Pre-vaccination	Post-vaccination	Pre-vaccination	Post-vaccination
*Overall*	Number of relationships	399	414	93	100
	Number of isolates	867	852	276	269
*Components*	Number of components^a^	243	243	74	74
	Size of main component	14	16	5	10
	Size of components				
	N = 14	1	1	0	0
	N = 10	1	1	0	1
	N = 9	2	2	0	0
	N = 8	2	2	0	0
	N = 7	2	3	0	0
	N = 6	3	3	0	0
	N = 5	11	9	1	1
	N = 4	7	8	4	4
	N = 3	39	39	6	5
	N = 2	175	174	63	63
	Average component size^a^	2.63	2.70	2.23	2.32
*Centrality and centralization*	Degree centrality (valued) – mean (SD)	1.53 (2.43)	1.56 (2.46)	1.77 (3.06)	1.78 (3.06)
	Degree centrality (binary) – mean (SD)	0.53 (0.80)	0.55 (0.82)	0.42 (0.59)	0.45 (0.63)
	Centralization (valued)	0.43	0.43	0.20	0.20
	Centralization (binary)	0.24	0.24	0.59	0.58
*Cohesion*	Transitivity	0.69%	0.63%	10.0%	7.1%
	Number of 2-cores	2	2	2	2
	Density (valued)	0.9904^b^	0.9970^b^	0.0067^c^	0.0069^c^
	Density (binary)	0.00035^d^	0.00037^d^	0.0019^e^	0.0020^e^
	Diameter	4	8	3	6

SD: standard deviation.

a Excluding isolates.

b Difference was no statistically significant (p = 0.356).

c Difference was not statistically significant (p = 0.139).

d Difference was statistically significant (p<0.001).

e Difference was statistically significant (p = 0.019).

Risk compensation resulted in a *decrease* in transitivity (10% to 7% in the sociometric network). The decrease in transitivity was likely due to the fact that individuals drawn into the network through risk compensation were not connected to other members of the network, creating more triads that did not exhibit closure. The number of 2-cores remained constant across the pre- and post-vaccination networks, but a 6% increase in density, from 0.00035 to 0.00037 based on binary data, was observed (p<0.001). As shown in Table 2, similar patterns were present when the analyses were restricted to the sociometric network.

## Discussion

Risk compensation in this sample was relatively uncommon; only 4% reported an intention to decrease condom use with a partner and 1% to increase sharing injection equipment if they, their partner, or they *and* their partner received an HIV vaccine. Risk compensation in the form of initiating sexual and/or equipment sharing with new partners was similarly rare (1%). The percentage of participants reporting an intention to risk compensate if given a highly efficacious vaccine is nearly half that reported in a study conducted among high-risk individuals recruited from clinics, syringe exchange programs, and Latino community-based organizations in Los Angeles [6] and one-fourth that reported among men who have sex with men, African American women, and drug users in Atlanta [39] and people who inject drugs from Philadelphia [55]. Comparisons between the current study and those referenced above should be made with caution given the vastly different contexts, specifications of efficacy (e.g., 50% and 99% [6], no specification [39], [55]), and risk compensation assessment (i.e. global measures of anticipated behavior change vs. risk compensation intent considered on a partner-by-partner basis). Most importantly, the contrast between the present study's findings and those from other settings should be considered in light of differences in community HIV prevalence, which is low in the present study's target community [56]. Thus, risk compensation intent may have been less prevalent in the current study due to low perceived risk for HIV acquisition (i.e., behavioral inhibition due to perceived HIV risk is currently low, thus behavioral disinhibition may be less applicable).

To our knowledge, the current study is the first to explore risk compensation under three vaccination scenarios: vaccination of self, partner, and self *and* partner. Interestingly, levels of risk compensation under the partner-vaccination scenario were nearly identical to that under personal vaccination. Previous research has generally assumed that risk compensation would be initiated by the vaccine recipients (i.e. by asking respondents about his/her intent to increase risk behavior if he/she was vaccinated, but not about their likelihood of changing behavior in response to partners' vaccination [6], [37], [39]), but this study provides evidence that partners of recipients may also initiate increased risk behavior. This dynamic is important to explore in future HIV vaccine acceptability research and in the context of HIV vaccine clinical trials.

While the individual-level data are valuable, only under examination at the dyadic level do the complex dynamics of risk compensation become apparent. More than 500 sexual partnerships and nearly 70 equipment-sharing relationships were reported by the drug users enrolled in this study. Intent to engage in sexual risk compensation was reported for 27 relationships, and intent to increase equipment sharing was reported for three. Thus, the 24 individuals who intended to increase their risk behavior would actually put 35 individuals in the network at increased risk for HIV transmission.

It is also important to note that in 5% of sexual relationships, condom use was anticipated to *increase* following HIV vaccination. This finding is corroborated by previous research reporting decreases in sexual risk behavior among participants enrolled in HIV vaccine clinical trials [15]–[17]. The potential for decreased risk behavior is important given evidence from simulation studies suggesting that to achieve maximal impact with a partially effective vaccine, vaccine uptake must be coupled with behavioral risk reduction [40], [41]. From the dyadic level, it is important to note that most of the relationships for which there was intended risk reduction currently involved *no* condom use. Thus, unless the couple decided to begin abstaining from unprotected sex completely, the impact of behavioral risk reduction would result in minimal change in HIV risk for first- and second-order partners.

Individual- and dyad-level changes in risk behavior can only be fully understood in the context of the larger social network in which high-risk individuals are embedded. The present study provides preliminary evidence that risk compensation could affect the connectivity of risk networks. For example, the density of the risk network constructed on the basis of participants' risk compensation intentions (i.e., accounting for new ties due to anticipated increases in risk behavior *and* lost ties due to intended decreases in risk behavior) was significantly greater than that of the current risk network. The structural changes observed were only slight, but conceptually important. Future studies involving network modeling will be needed to estimate the impact of these changes on epidemic potential. The findings of the present study will be valuable to the parameterization of these network models.

Generalization of the study's findings should be made with caution and in light of its limitations. The measure of risk compensation was based on intention; intended behavior change may or may not correspond with patterns of future risk behavior [57], [58]. Additionally, the self-reported behavioral data were subject to social desirability and recall bias. Also, because participants were aware that the survey would request additional information about each named alter, participants may have been reluctant to provide names on the two open-ended questions seeking information about new partners with whom they would initiate a risk relationship in response to receiving a vaccine; thus, the number of new partnerships may have been underestimated. Due to time-constraints and extended length of the questionnaire posed by the number of alter-specific questions, specific types of sexual activity (e.g. vaginal, anal, oral) and measures of risk compensation in the context of hypothetical pre-exposure prophylaxis use and varying levels of vaccine efficacy were unable to be assessed. The specification of efficacy-level in the questionnaire was important in standardizing responses, as a vaguely worded item would create undue variance in responses with conceptualizations of efficacy varying among respondents. The 90% efficacy level was chosen as it allowed for the most conservative estimate of risk compensation (i.e., a level that would greatly mitigate perceived acquisition risks and potentially lead to near maximum compensation behaviors).

This study took place among a unique population of rural drug users who live in a region with low HIV incidence [56]. In fact, none of the named alters were perceived to be HIV positive. This could have influenced estimates of risk compensation intent, as vaccination would be expected to precipitate less behavior change in relationships posing less risk. While these factors may dampen the generalizability of the findings to settings with higher HIV burden, the conceptual and methodological approach certainly remains applicable to research in other settings. Finally, though they would have provided valuable insight, contextual data on the circumstances and motivations surrounding risk compensation were not collected. In the future, qualitative approaches are needed to fully explore the complexity of anticipated behavior change in response to HIV vaccination.

This study provides a methodological framework in which to examine anticipated risk compensation in future HIV vaccine preparedness cohorts and to examine the network-level impact of behavioral change in future HIV vaccine clinical trials. In future research, risk compensation measures should assess not only *if* people risk compensate, but also *with whom* they risk compensate. This study also suggests that network-level change be considered in the parameterization of mathematical models projecting the impact of risk compensation on the success of future HIV vaccines. Finally, the findings from this study on the infrequency of intended risk compensation, particularly that related to syringe sharing, are encouraging and underscore the positive potential impact of a future HIV vaccine.
